# Relationship of Early Spontaneous Type V Blood Pressure Fluctuation after Thrombolysis in Acute Cerebral Infarction Patients and the Prognosis

**DOI:** 10.1038/srep27656

**Published:** 2016-06-09

**Authors:** Lian Zuo, Ting Wan, Xiahong Xu, Feifeng Liu, Changsong Li, Ying Li, Yue Zhang, Jing Zhang, Huan Bao, Gang Li

**Affiliations:** 1 Department of Neurology, East Hospital, Tongji University School of Medicine, Shanghai, China; 2 Department of Neurology, Shanghai East Hospital, Dalian Medical University, Liaoning Province, China

## Abstract

We examined the relationship between an early spontaneous type V blood pressure fluctuation and the post-thrombolysis prognosis of patients with acute cerebral infarction. Patients were admitted consecutively. All patients were categorized into the type V blood pressure fluctuation group or non-type V blood pressure group. Their blood pressure was monitored before thrombolysis and until 6 h after thrombolysis. Baseline data and clinical outcomes were compared. Of 170 patients, 43 (25.2%) had an early type V blood pressure fluctuation. The National Institute of Health Stroke Scale (NIHSS) score before thrombolysis and 24 h after thrombolysis, and the modified Rankin scale score at 90 days differed significantly between the two groups (P < 0.05). Multiple logistic regression analysis showed that an unfavorable prognosis at 3 months was associated with the NIHSS score before thrombolysis (P = 0.000) but probably not with this blood pressure fluctuation (P = 0.058). An early spontaneous type V blood pressure fluctuation is common in patients with acute cerebral infarction who received venous thrombolysis, especially if they have a higher NIHSS score before thrombolysis. The type V blood pressure fluctuation may not influence patients’ prognosis; however, this needs to be confirmed in future trials.

Acute cerebral infarction is a neurological emergency usually with an elevated blood pressure (BP) during the early period. There are several possible causes such as the inadequate treatment of hypertension, undiagnosed hypertension, stress reaction caused by an activated neuroendocrine system, an impaired cerebral autonomic regulatory center, and elevated intracranial pressure^1^. Cerebral tissue perfusion is dependent on circulatory BP, and cerebral autonomic regulation is often impaired during acute cerebral infarction. Thus, early antihypertensive therapy can decrease blood perfusion in the ischemic penumbra and enlarge the infarction area^2^. Moreover, excessively high BP can exacerbate cerebral edema and cause hemorrhagic transformation^3^. There is still no guideline on when to start antihypertensive therapy during the acute stage of cerebral infarction and which treatment protocol is the best for managing BP^4^,^5^,^6^,^7^.

Currently, early intravenous thrombolysis is an effective therapy for acute cerebral infarction^8^,^9^. Elevated systolic BP before thrombolysis can decrease revascularization rate, increase the risk of hemorrhagic transformation^10^. According to the American Heart Association/American Stroke Association’s guidelines, the recommended BP is ≤180/105 mmHg 24 h after intravenous thrombolysis with recombinant tissue plasminogen activator (rtPA)^11^, but there is no optimal BP level when the BP is <180/105 mmHg. Post-thrombolysis blood pressure management in patients with acute cerebral infarction has been a research hotspot. Yong *et al*^12^ reviewed patients’ data in ECASS-II and found that increased systolic blood pressure within 24 hours after thrombolysis was associated with a poor prognosis. SITS-ISTR (Safe Implementation of Thrombolysis in Stroke -International Stroke Thrombolysis Register) study found that the average systolic blood pressure 2 hours and 24 hours post-thrombolysis had a "U-type" relationship with the prognosis in patients with acute cerebral infarction, with the best prognosis seen in patient with an average systolic blood pressure 141–150 mmHg 24 hours after thrombolysis^13^. While SAMURAI (Stroke Acute Management with Urgent Risk-factor Assessment and Improvement) rt-PA Registry experiment confirmed that blood pressure variability within 24 hours after thrombolysis was positively correlated with hemorrhagic transformation and death^14^. There have also been reports that an obvious decrease of systolic BP after 24 h post-thrombolysis is associated with a good outcome 90 days after thrombolysis^15^.

During clinical routine workups, we note that spontaneous BP fluctuation, which is the recovery after an abrupt decrease of BP, is exhibited early after intravenous thrombolysis in some patients with acute cerebral infarction. This is an interesting phenomenon which has been reported previously^12^,^14^, but the clinical significance of this phenomenon remains to be explored, and no correlation of this phenomenon with diagnosis or prognosis of acute cerebral infarction has been summarized or established so far. In the current study, we want to know whether this fluctuation of BP can influence these patients’ prognosis and whether it requires clinical intervention. BP changes of patients with acute cerebral infarction were observed prospectively after intravenous treatment with rtPA, and follow-up was continued for 90 days to clarify this phenomenon and determine the relationship between the fluctuation and patients’ prognosis.

## Subjects and Methods

### Patient population

From February 2013 to March 2015, patients with acute cerebral infarction who received intravenous rtPA were admitted consecutively in the Department of Neurology at East Hospital. Inclusion criteria were as follows: (1) patients with acute cerebral infarction during the observation period confirmed with cranial computed tomography or magnetic resonance imaging^16^, and (2) those who received venous thrombolysis with an rtPA. Exclusion criteria were (1) patients who received antihypertensive agents or vasopressors before thrombolysis or within 6 h after thrombolysis; (2) those who received artery intervention after venous thrombolysis; and (3) patients misdiagnosed as acute ischemic stroke.

We defined the BP fluctuation as a type V fluctuation as follows: (1) the patients’ systolic BP decreases abruptly from peak pressure following partial recovery; (2) peak BP is the highest systolic BP during the first 15 min since the beginning of thrombolysis; (3) amplitude of systolic BP decreases over 20% of its peak pressure; and (4) recovery amplitude of systolic BP is over 10% of its peak pressure. The patients’ BPs were monitored before thrombolysis and until 6 h after thrombolysis, with the main observational index of supine brachial systolic BP. The BP was recorded every 15 min during the first 2 h since thrombolysis was started, and then it was recorded every half hour during the next 2–6 h. According to whether type V BP fluctuation appeared within 6 h after thrombolysis, all patients were categorized into the type V BP fluctuation group (type V BP group) or non-type V BP group (non-type V BP group). The patients’ sex; age; prior history of hypertension, diabetes mellitus, atrial fibrillation, ischemic stroke, and smoking; onset to thrombolysis time; and the National Institute of Health Stroke Scale (NIHSS) score (scores range from 0–42, with higher scores indicating increasing severity) were evaluated before thrombolysis, and they were followed up for 3 months. The thrombolysis dosage was 0.9 mg/kg per body weight.

The study was carried out in accordance with the guidelines, which was approved by the local institutional review board at East Hospital, Tongji University, and the informed consent was obtained from the patients.

### Follow-ups

The NIHSS scores 24 h after thrombolysis and 2 wk after thrombolysis were assessed. The patients’ outcomes were assessed 90 days after stroke using the modified Rankin scale (mRS, 0–6; 0–1 indicates a favorable prognosis, whereas 2–6 indicates an unfavorable prognosis). The other endpoints included recurrent stroke, death, and lost to follow-up. All clinical assessments were made by experienced neurologists.

### Statistical analysis

Data were expressed as 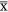 ± s or the median, and analyzed using SPSS 21.0 (IBM Corp.). The measurement data were analyzed using Student’s t test or the Mann-Whitney test. The chi square test or Fisher exact test was used for numerical data. The prognosis at 90 days was estimated using multivariate logistic regression analysis in which the mRS scores were regressed on the following baseline covariates that were thought to be associated with the outcomes of interest, i.e., patients’ age, sex, prior history, onset to thrombolysis time, and NIHSS score before thrombolysis, etc. P < 0.05 indicated statistical significance.

## Results

One hundred ninety-three patients underwent venous thrombolysis during the study; of those, 15 took antihypertensive agents because their BP was over 185/110 mmHg before thrombolysis or during the first 6 h of thrombolysis, 2 received vasopressors, 3 received percutaneous intervention therapy after thrombolysis, and 3 misdiagnosed as cerebral infarction were all excluded.

**Figure 1:**
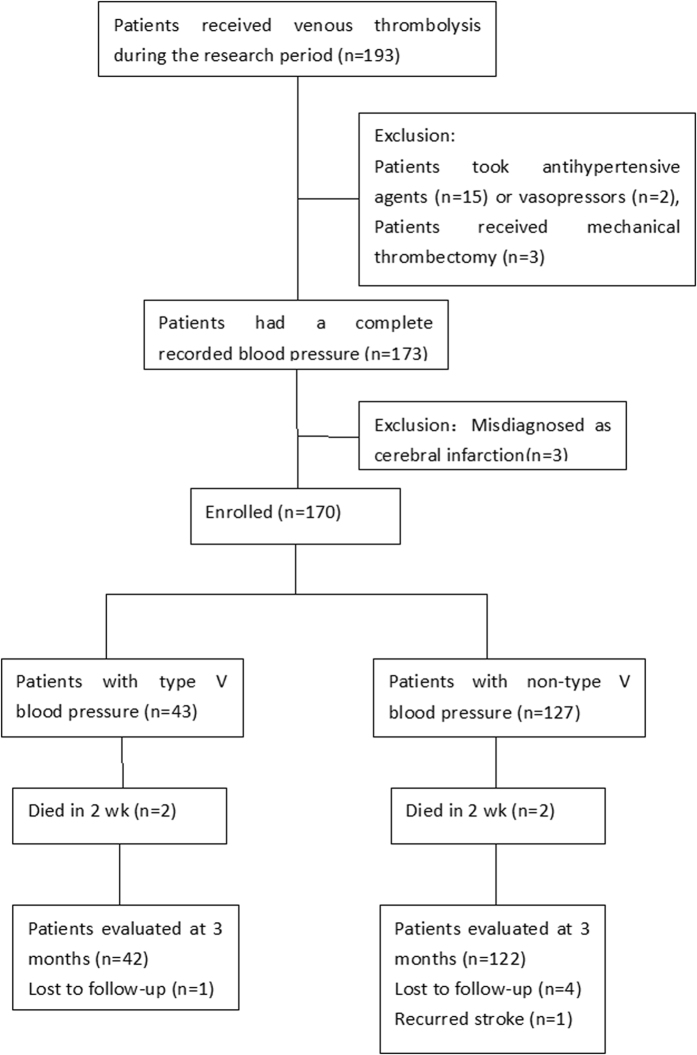
Study flow chart.

**Figure 2:**
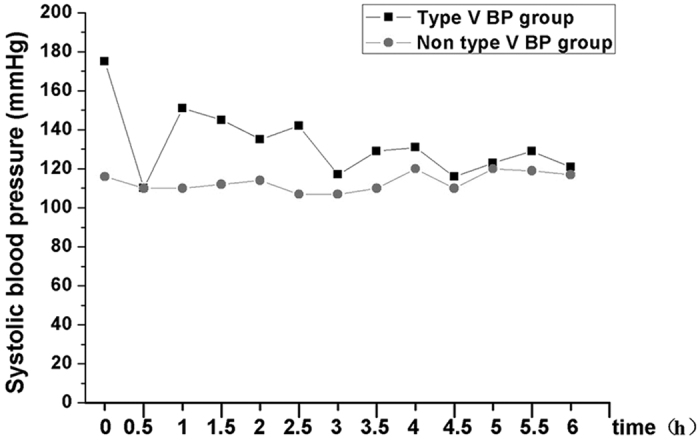
A patient’s systolic blood pressure (BP) in the type V BP group during the first 6 h (60-year-old man. National Institute of Health Stroke Scale [NIHSS] score = 9 before thrombolysis) and a patient’s systolic BP in the non-type V BP group during the first 6 h (25-year-old man, NIHSS score = 3 before thrombolysis).

Thus, 170 patients were enrolled; 101 (59.4%) were men and 69 (40.6%) were women. They were 25–92 years old with an average age of 65.5 ± 10.8 years. All enrolled patients were categorized into the type V BP group (43 patients [25.3%]) or non-type V BP group (127 patients [74.7%]). One hundred sixteen (68.2%) patients had an NIHSS score ≤8 before thrombolysis, whereas 54 (31.8%) had an NIHSS score >8. The onset-treatment time ranged from 40–320 min with an average time of 182 ± 53 min. Finally, 164 patients completed the follow-up (type V BP group: 42 patients, non-type V BP group: 122 patients). Five patients were lost to follow-up, 1 from the type V BP group (2.3%) and 4 from non-type V BP group (3.1%), one patient got recurrent stroke (Fig. 1).Examples of the systolic BP in patients from the two groups during first 6 h are shown in Fig. 2.

### Baseline data

There was no significant difference in sex, age, hypertension, diabetes mellitus, atrial fibrillation, ischemic stroke, smoking, and onset-treatment time between the two groups. There was a statistical difference in the NIHSS score before thrombolysis between the two groups (P < 0.001) (Table 1).

### Clinical outcomes

In the first 2 wk, 4 of 170 patients died, 2 of whom died of cerebral edema, 1 of pulmonary embolism, and 1 of pulmonary infection. During the 3 months, 1 patient had a recurrent cerebral infarction, 5 were lost to follow-up, and there were no new deaths. There was a statistical significance in the difference of the NIHSS score 24 h after thrombolysis (P < 0.05) but no statistical significance in the difference of the NIHSS score 2 wk after thrombolysis. Additionally, there was statistical significance in the difference of the mRS score during the following 3 month after thrombolysis (P < 0.05) (Tables 2 and 3).

### Prognosis

**Figure 3:**
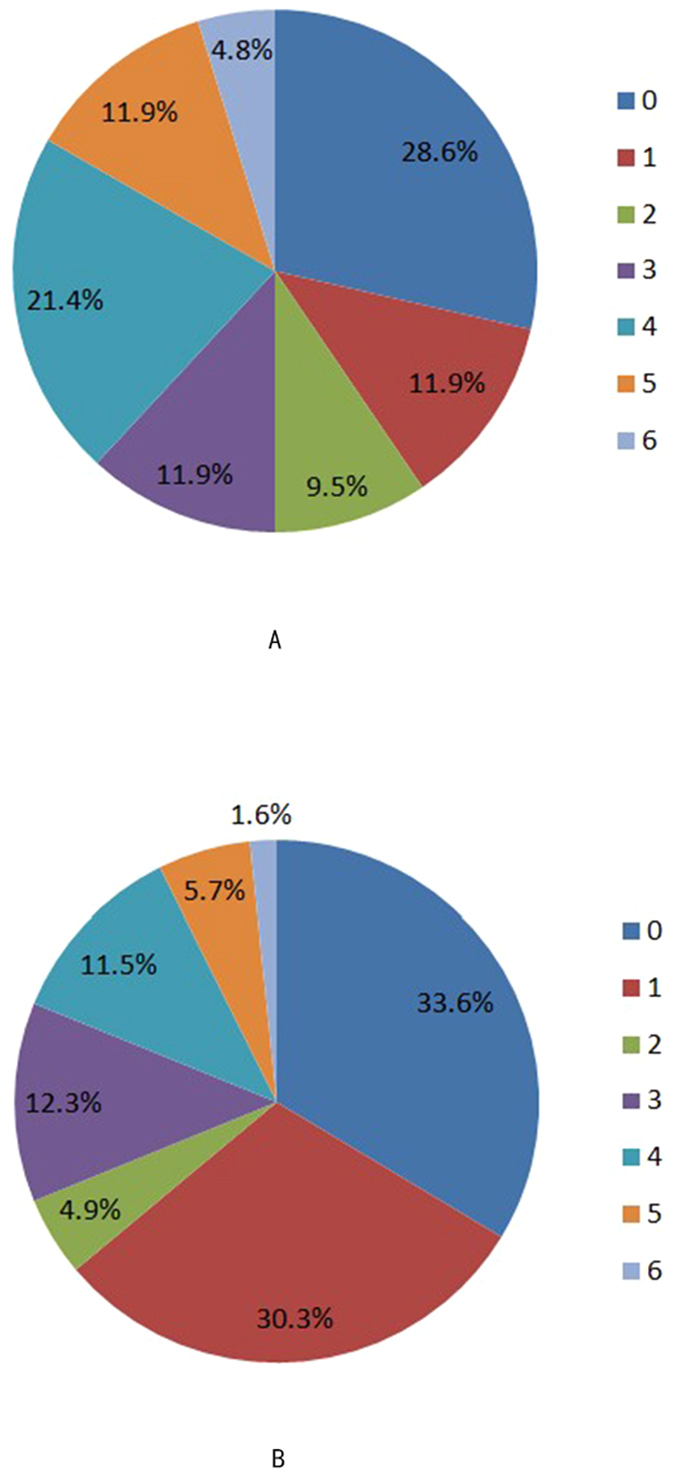
(**A**) Distribution of the modified Rankin scale scores of patients in the type V blood pressure group over the 3 months. (**B)** Distribution of the modified Rankin scale scores in patients in the non-type V blood pressure group over the 3 months.

The prognosis of patients was defined as favorable or unfavorable according to the mRS scores. Distributions of the modified Rankin scale scores of patients in two groups are shown in Fig. 3. The correlation analysis showed that a history of atrial fibrillation was associated with NIHSS scores before thrombolysis so the history of atrial fibrillation was excluded from logistic regression. The results of multivariate logistic regression analysis showed that NIHSS scores >8 before thrombolysis were associated with a 5.3-fold increase in the risk for an unfavorable prognosis (P = 0.000; odds ratio [OR]: 5.264; 95% confidence interval [CI]: 2.468–11.225), and type V BP fluctuation was not associated with an unfavorable prognosis (P = 0.058; OR: 2.188; 95% CI: 0.974–4.915) (Table 3).

## Discussion

Intravenous rtPA was approved for treating acute cerebral infarction by the United States Food and Drug Administration in 1996, and it is the only drug for thrombolysis confirmed by clinical trials performed in the United States and Europe that is recommended for ischemic stroke. This drug is a type of fibrin dissolved suppository that can selectively combine fibrin in the surface of a thrombus and develop a fibrin complex to activate plasminogen to transform into plasmin, which can dissolve fibrin and restore blood flow to rescue neurons in ischemic penumbra and accordingly restore neurological function^8^.

As mentioned earlier, the increased systolic blood pressure or systolic blood pressure variation in acute cerebral infarction patients within 24 hours after the intravenous thrombolytic therapy was associated with a poor prognosis, so close monitoring and strict management of blood pressure after thrombolysis has been widely deemed necessary. It was confirmed in our study that spontaneous type V BP fluctuation had occurred early after venous thrombolysis in 25% of patients with acute cerebral infarction. Further analysis indicated that the NIHSS score was higher in the type V BP group than in the non-type V BP group before thrombolysis, and it was significantly different (P < 0.001). Thus, it is speculated that a spontaneous type V blood pressure fluctuation occurs more commonly in patients with a higher NIHSS score before thrombolysis, which usually indicates a more severe neurologic defect. The “V type” blood pressure fluctuation phenomenon in this study was different from previously reported post-thrombolytic blood pressure variability in terms of calculation methods and definitions^12^,^14^.

The type V BP fluctuation for acute cerebral infarction after venous thrombolysis has been reported in the past, but its clinical significance or whether clinical intervention was needed were not clear. Our current study confirmed the existence of such phenomenon, furthermore, our result suggested that this BP fluctuation did not influence patients’ prognosis, and it probably does not require clinical intervention.

The possible mechanism of a type V BP fluctuation for acute cerebral infarction after venous thrombolysis may be that there is an autonomic regulation of cerebral blood flow in patients with ischemic stroke. Temporary systolic BP elevation occurs in 80% of patients with acute cerebral infarction to raise the cerebral perfusion pressure^17^. When occlusive vessels recanalize after thrombolysis, it is unnecessary for those patients to maintain a higher systolic BP. As a result, the BP decreases^18^. Through retrospective analysis of 17 patients with acute cerebral infarction who also received intravenous thrombolysis treatment, Nagaraja *et al* found that a decrease of systolic blood pressure ≥20 mmHg 24 h post-thrombolysis than that of pre-thrombolysis accompanied by neurological improvement may indicate revascularization^19^, and there were reports confirming that a sudden drop of systolic blood pressure (≥20 mmHg)within 2 h post-thrombolysis in patients with cerebral vascular obstruction indicated revascularization^20^. However, we think this mechanism does not explain why the BP increases again. Besides, intravenous rtPA can activate the bradykinin pathway to activate a complement cascade, which increases C4a and C5a to facilitate vasoactive substances released from mast cells and basophilic granulocytes. Thus, blood vessels were dilated causing the BP to decrease, which can happen about 90 min after drug administration^21^,^22^. The BP recovers because the half-life of an rtPA is only 15–20 min, and this may be the reason for the type V BP fluctuation, which still needs to be further investigated.

During follow-up, there was a difference in the 24 h NIHSS score but no obvious difference at 2 wk between the two groups, which indicated that the spontaneous type V BP fluctuation had no obvious influence on patients’ short-term prognosis. However, after the 3-month follow-up, we found that 40.5% of patients in the type V BP group had a favorable prognosis (mRS score 0–1), and 63.9% of patients in the non-type V BP group had a favorable prognosis, which indicated that patients with the early spontaneous type V BP fluctuation (i.e., within 6 h after thrombolysis) may have had an even worse long-term prognosis. Thus, the prognosis at 3 months was further evaluated using multivariate logistic analysis in which the mRS scores were regressed on the baseline covariates, and we found that only NIHSS scores >8 before thrombolysis were associated with an unfavorable prognosis (P = 0.000) but not the type V BP fluctuation (P = 0.058).

Currently there is no definitive conclusion on the reasonable range of blood pressure management in acute cerebral infarction after thrombolysis, retrospective data analysis suggested that the prognosis was better in patients with a mean systolic blood pressure of 140–150 mmHg within 24 hours after thrombolysis^13^. In the ongoing ENCHANTED trial^23^, patients with acute cerebral infarction undergoing thrombolytic therapy were divided into early and aggressive management of blood pressure group (SBP between 130–140 mmHg) and according-to-guideline blood pressure management group (SBP ≤ 180 mmHg) to observe the outcomes. The results of the ENCHANTED trial will contribute to the development of post-thrombolysis blood pressure management protocol.

There are some limitations to our study. It was a single center study with a small sample and relatively simple observational index (only the systolic BP). Besides, the observation time was only within 6 h after thrombolysis. During the study, some patients with severely impaired neurological function had a BP > 180/110 mmHg. These patients were given antihypertensive agents for intravenous thrombolysis, and they were finally excluded (15 cases) from this study. Thus, the enrolled patients did not represent all patients with cerebral infarction at our hospital during the study, which may have caused some bias (i.e., some severe patients were excluded). Lastly, the loss of follow-up caused by removal and contact information changes for some of the patients may have influenced the results.

## Conclusion

In conclusion, we confirmed that a spontaneous type V BP fluctuation occurs early after intravenous thrombolysis in some patients with acute cerebral infarction, especially in those with a higher NIHSS score. This type V BP fluctuation may not influence patients’ prognosis, and it probably does not require clinical intervention; however, larger multicenter clinical observation trials with larger sample size are needed to clarify whether this type V BP fluctuation influences the prognosis of patients and whether it requires intervention. The practical implications of this study may lead to further investigations of this topic, assisting in the development of novel guidelines in the treatment of acute cerebral infarction.

## Additional Information

**How to cite this article**: Zuo, L. *et al* Relationship of Early Spontaneous Type V Blood Pressure Fluctuation After Thrombolysis in Acute Cerebral Infarction Patients and the Prognosis. *Sci. Rep.* **6**, 27656; doi: 10.1038/srep27656 (2016).

## Supplementary Material

Supplementary Information 1

Supplementary Information 2

## Figures and Tables

**Table 1:** Baseline Characteristics of the Study Participants.

Characteristic	Type V BP group (n = 43)	Non-type V BP group (n = 127)	P value
Male sex, no. (%)	25 (58.1)	76 (59.8)	0.844
Age, mean (SD), years	67 (10)	65 (11)	1.000
Hypertension, no. (%)	33 (76.7)	95 (74.8)	0.799
Diabetes mellitus, no. (%)	9 (20.9)	37 (29.1)	0.295
Atrial fibrillation, no. (%)	13 (30.2)	29 (22.8)	0.331
Ischemic stroke, no. (%)	4 (9.3)	13 (10.2)	1.000
Smoker, no. (%)	19 (44.2)	57 (44.9)	0.937
Onset to thrombolysis time, mean (SD), min	187 (51)	181 (54)	0.496
NIHSS score before thrombolysis, median (min, max)	8 (1, 24)	5 (0, 21)	<0.001*

^*^P < 0.05.

BP, blood pressure; SD, standard deviation; NIHSS, National Institute of Stroke Scale.

**Table 2:** Clinical Outcomes of the Type V BP Group and Non-type V BP Group.

	Type V BP group (n = 43)	Non-type V BP group (n = 127)	P value
NIHSS scores 24 h after thrombolysis, median (min, max)	4.5 (0, 23)	3 (0, 30)	0.045*
NIHSS scores 2 wk after thrombolysis, median (min, max)	2 (0, 24)	2 (0, 32)	0.413
Deaths, no. (%)	2 (4.7)	2 (1.6)	0.265
Lost to follow-up during the 3 months, no. (%)	1 (2.3)	4 (3.1)	1.000
Newly developed stroke during the 3 months, no. (%)	0 (0)	1 (0.8)	1.000
mRs scores (0–1) during the 3 months, no. (%)	17 (39.5)	78 (61.4)	0.016*

^*^P < 0.05.

BP, blood pressure; NIHSS, National Institute of Stroke Scale; mRs, modified Ranking scale.

**Table 3:** Multivariate Logistic Regression Analysis of Determinants of an Unfavorable Prognosis.

Covariate	B	SE	OR	95% CI	P value
Sex	−0.389	0.470	0.677	0.269–1.703	0.408
Hypertension	0.826	0.444	2.285	0.957–5.457	0.063
Diabetes mellitus	0.003	0.419	1.003	0.441–2.281	0.995
Age >80 years	0.087	1.011	1.091	0.150–7.913	0.931
Smoker	−0.720	0.483	0.487	0.189–1.254	0.136
Ischemic stroke	0.768	0.616	2.156	0.645–7.207	0.212
Onset to thrombolysis time >3 h	−0.098	0.362	0.907	0.446–1.842	0.786
NIHSS score before thrombolysis >8	1.661	0.386	5.264	2.468–11.225	0.000*
Type V BP fluctuation	0.783	0.413	2.188	0.974–4.915	0.058

^1^P < 0.05.

B, regression coefficient; SE, standard error; OR, odds ratio; CI, confidence interval; BP, blood pressure; NIHSS, National Institute of Stroke Scale.
