# Supplementary figures and images for: Relationship of Early Spontaneous Type V Blood Pressure Fluctuation after Thrombolysis in Acute Cerebral Infarction Patients and the Prognosis
Source: Sci Rep. 2016 Jun 9;6:27656. doi: 10.1038/srep27656 (PMC4899732; doi:10.1038/srep27656)

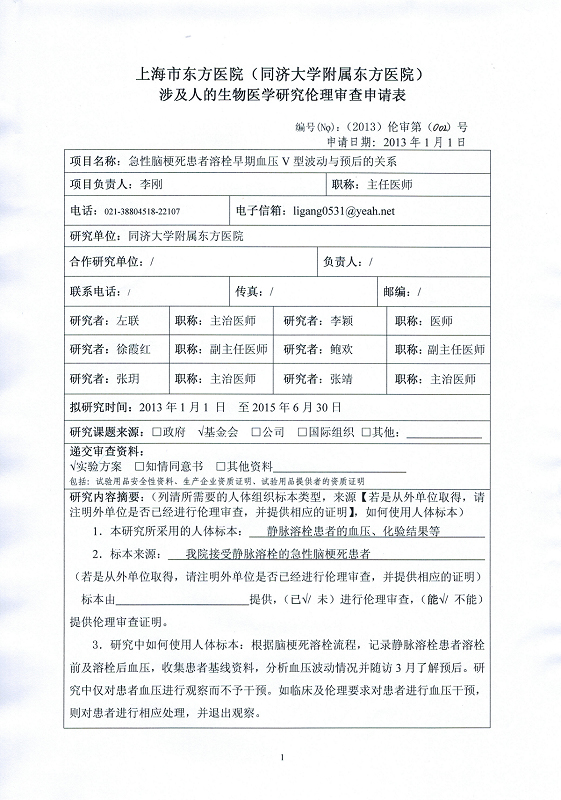

Supplement: Supplementary Information 1 [file srep27656-s1.jpg]

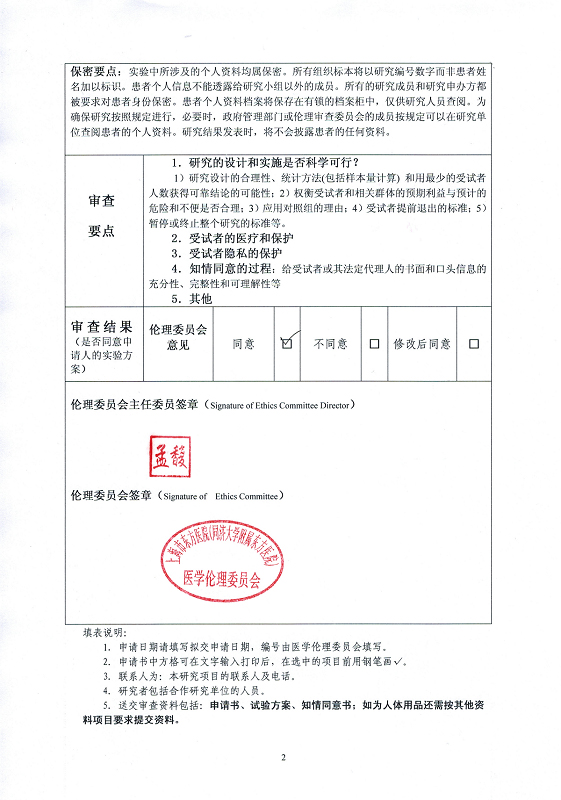

Supplement: Supplementary Information 2 [file srep27656-s2.jpg]
